# Multi-plane echocardiographic assessment of right ventricular function in adults with repaired Tetralogy of Fallot

**DOI:** 10.1007/s10554-021-02273-5

**Published:** 2021-05-18

**Authors:** Daniel J. Bowen, An M. van Berendoncks, Jackie S. McGhie, Jolien W. Roos-Hesselink, Annemien E. van den Bosch

**Affiliations:** 1 Department of Cardiology, Erasmus MC Thorax Center, Rotterdam, The Netherlands; 2 Department of Cardiology, Antwerp University Hospital, Antwerp, Belgium; 3 University of Antwerp, Antwerp, Belgium

**Keywords:** Tetralogy of Fallot, Echocardiography, Right ventricle, Right ventricular strain, Multi-plane

## Abstract

In patients with repaired Tetralogy of Fallot (ToF), detailed assessment of right ventricular (RV) function is important for management and timing of possible pulmonary valve re-intervention. The aim of this study was to evaluate RV function using two-dimensional multi-plane echocardiography (2D MPE), a novel four-wall imaging method obtained from one apical acoustic window utilising electronic plane rotation. In sixty-two ToF patients (aged - 28 [22, 39] years, 65% male), systolic function of four different RV walls (lateral, anterior, inferior and inferior coronal) were evaluated using MPE. Tricuspid annular plane systolic excursion (TAPSE), tricuspid annular peak systolic velocity (RV-S′) and RV wall longitudinal strain (RV-LS) measurements were compared with those of matched healthy individuals. 2D MPE measurements were highly feasible across the four RV walls (93.5–100% for TAPSE/S′; 66.1–95.1% for RVLS) and could be performed more reliably than 3D RV ejection fraction (RVEF − 56.5%). All functional values were significantly reduced when compared to the control group (p < 0.001). Higher RV-LS values were seen in the lateral (− 17.8 ± 4.5%) and inferior (− 17.8 ± 4.2%) walls compared to the anterior (− 15.9 ± 3.8%) and inferior coronal (− 15.1 ± 3.9%) walls. 3D RVEF correlated strongest with RV-LS values from the lateral (r − 0.50; p = 0.002) and anterior walls (r − 0.74; p < 0.001) and furthermore the four-wall average (r − 0.57; p = 0.001). 2D MPE evaluation of the RV is highly feasible in ToF patients. This novel method provides new insights into regional RV wall function, enabling a more comprehensive and quantitative approach to RV assessment in daily clinical practice.

**Supplementary Information:**

The online version contains supplementary material available at 10.1007/s10554-021-02273-5.

## Introduction

Tetralogy of Fallot (ToF) is one of the most prevalent congenital heart diseases encountered in adulthood. Owing to successful intervention in infancy, patient survival and subsequent quality of life has been dramatically improved in this population [1]. Nevertheless, late cardiac complications, such as significant residual pulmonary regurgitation (PR), right ventricular (RV) dilatation and dysfunction, left ventricular (LV) dysfunction and arrhythmias are common [2]. As a result, many patients will undergo further intervention in the form of a surgical or percutaneous pulmonary valve replacement. However, the optimal timing of intervention remains controversial and the impact upon RV function is not fully understood [3]. RV dysfunction has been studied extensively in patients with repaired ToF and has been recognized as an important prognostic factor [1, 4]. Whilst two-dimensional trans-thoracic echocardiography (2D-TTE) is most widely used in congenital heart disease, this imaging modality has inherent limitations. Given the structural complexity of the RV, with inlet, outlet and apical regions, it is not possible to visualise the entire chamber from a single 2D-TTE acoustic window [5]. Furthermore, quantitative functional parameters are limited to one free wall region of the RV, namely the lateral wall. This is a limitation that may result in an over or under estimation of global RV function [6].

**Fig. 1:**
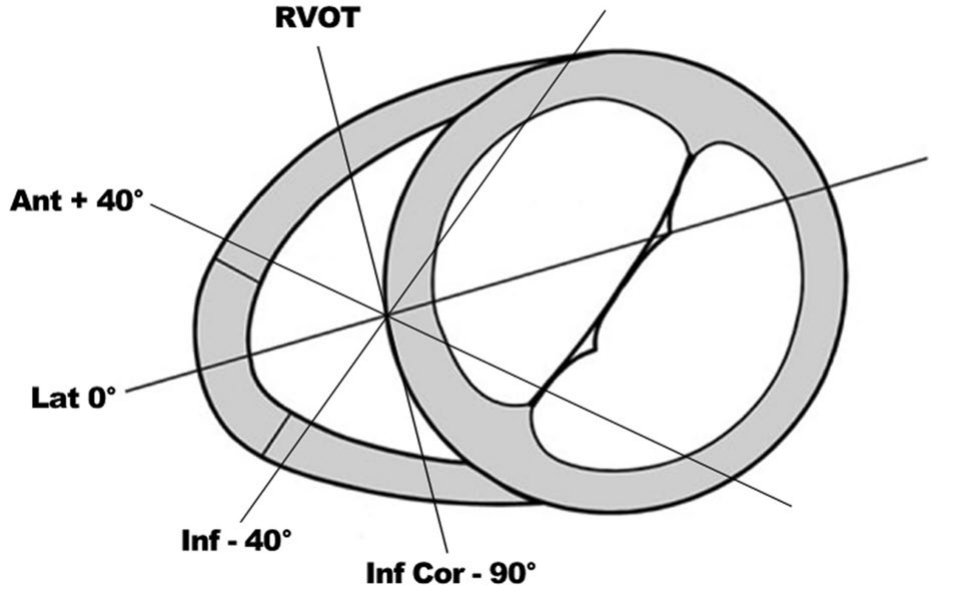
Multi-plane imaging of the right ventricle (RV) using 2D multi-plane echocardiography. Views obtained by electronic plane rotation around a single RV focused apical echocardiographic position. 0° rotation: lateral wall; + 40°: anterior wall; − 40°: inferior wall; − 90°: inferior wall coronal view also visualizing the right ventricular outflow tract

In order to address these drawbacks, our research group previously introduced a novel imaging approach utilising 2D multi-plane echocardiography (MPE). Whilst maintaining a fixed RV apical transducer position, four different RV walls based on anatomic landmarks (lateral, anterior, inferior and inferior coronal) (Fig. 1), can be imaged with a 3D ultrasound transducer using electronic plane rotation [7]. This new method, also known as iRotate mode, allows for a more detailed quantitative assessment of global and regional RV function than is presently performed using a standard multi-view 2D echocardiographic approach.

The aims of this study are (a) to evaluate the feasibility of this multi-plane method to quantify RV function in patients with repaired ToF and (b) to assess regional RV wall differences within this patient population as compared to those of age and gender matched healthy individuals.

## Methods

### Study population and echocardiographic acquisition

All ToF patients with a dilated RV seen routinely in the congenital cardiology outpatient clinic at our centre between 2014 and 2017 were considered for inclusion in this study. For adequate 2D MPE and 3D-TTE imaging to be performed, at least moderate to good echocardiographic image quality is required. An echo was excluded from the study if the entire RV free wall was poorly visualised in the focused apical four chamber view. Without clear delineation of RV endocardial borders, 2D MPE and 3D-TTE was adjudged unfeasible to perform. Screened individuals underwent detailed RV assessment by 2D multi-plane echocardiography, in addition to clinical evaluation and 12 lead electrocardiogram (ECG). All echocardiograms were performed by one echocardiographer (JSM) specialised in congenital echocardiography. Studies were acquired using an iE33 or EPIQ7 ultrasound system (Phillips Medical Systems, Amsterdam, The Netherlands) equipped with an X5-1 matrix array transducer (composed of 3040 elements with 1-5 MHz). All RV focused images were acquired at frame rates of ≥ 50 frames/s in order for longitudinal strain analysis to be performed. Real-time 3D-TTE was performed immediately after the 2D-TTE with the same ultrasound unit and transducer. A four- or six-beat full volume dataset (27 ± 6 vol/s, range 18–46) of the RV was acquired from the apical window during a single breath hold. To be able to compare the multi-plane and 3D RV values with a healthy population, we used a control group matched for age and gender. For this control group, self-declared healthy volunteers were prospectively recruited through advertisements, the details of which have been published previously [7]. The study was carried out according to the principles of the Declaration of Helsinki, was approved by the local medical ethics committee and written informed consent was obtained from all subjects.

### Conventional RV echocardiographic measurements

2D/3D echocardiographic parameters for left and right ventricular size and function were collected in addition to the grading of any valvular lesions. RV basal and longitudinal linear dimensions alongside fractional area change (FAC – calculated as end-diastolic area – end-systolic area/end-diastolic area × 100) were measured in the standard focused RV apical four chamber view conforming to international guidelines [8]. The degree of pulmonary regurgitation was defined quantitatively and semi-quantitatively using colour, pulsed and continuous wave Doppler assessment and graded as either less than ( <) or equal or greater than ( ≥) moderate in severity. A dense diastolic regurgitant flow signal on CW Doppler, wide jet width on colour Doppler, a short pressure half time measurement (< 100 ms) and/or presence of diastolic backflow in the main pulmonary artery and branches were seen as evidence of significant regurgitation [9].

### Right ventricular assessment by 2D multi-plane echocardiography.

**Fig. 2:**
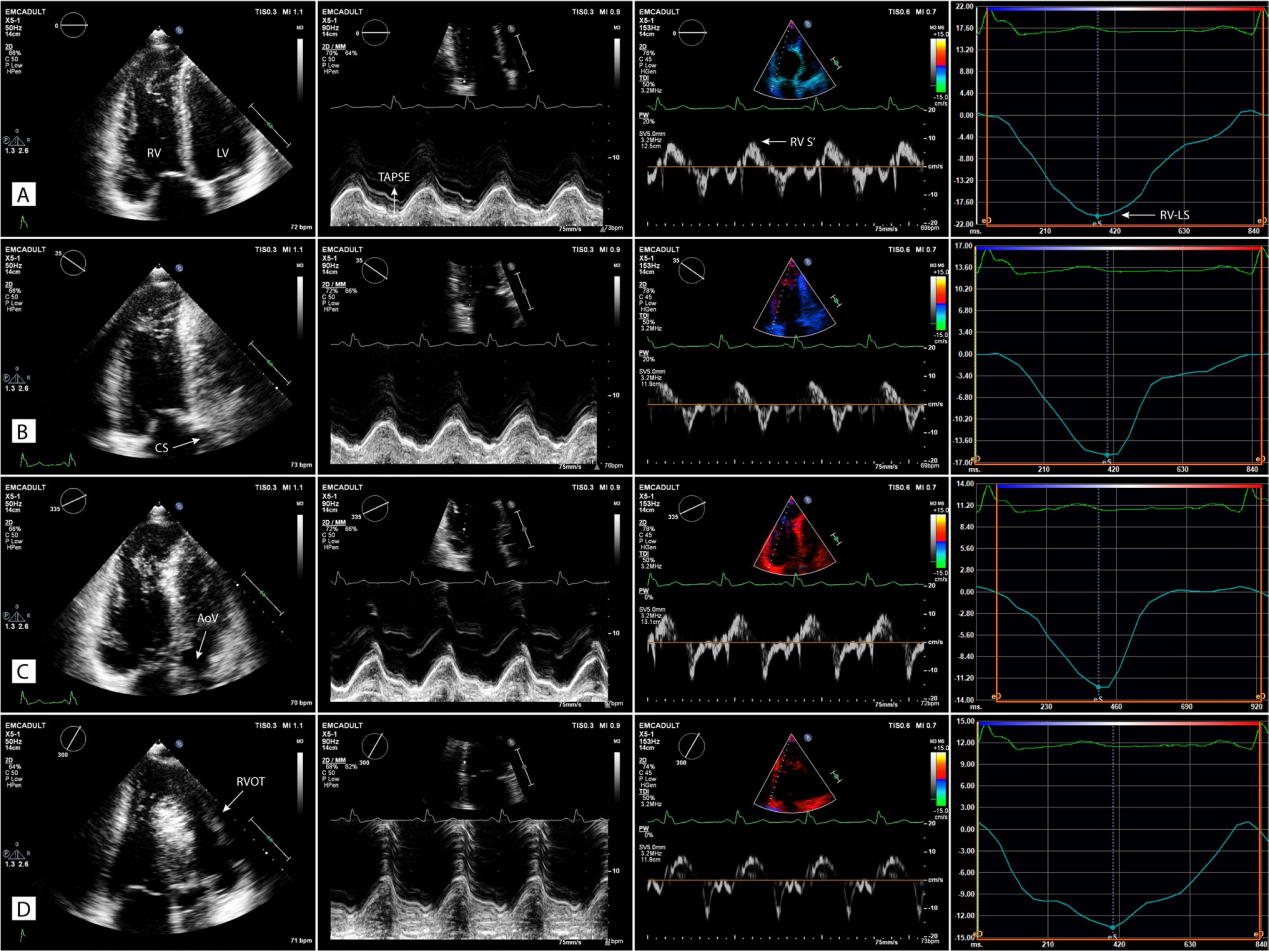
Echocardiographic images of the four multi-plane right ventricular (RV) views (A-D) with corresponding quantitative functional parameters of the respective RV walls (L-R panels). **A** Focused four chamber view (0°), lateral wall; **B** coronary sinus view (+ 40°), anterior wall; **C** aortic view (− 40°), inferior wall; **D** coronal view (− 90º), inferior wall and RVOT anterior wall. Second panel (centre left): tricuspid annular plane systolic excursion (TAPSE); third panel, (centre right): tricuspid annular peak systolic velocity (RV-S′); fourth panel (far right): RV wall longitudinal strain (RV-LS). *LV* left ventricle, *CS* coronary sinus; *AoV* aortic valve, *RVOT* right ventricular outflow tract

2D multi-plane echocardiographic assessment of the RV has been previously demonstrated by our centre and allows for multiple RV walls to be assessed from one echocardiographic position [6, 7]. To acquire the four additional RV views, a focused, non-foreshortened RV view is required with the RV apex and interventricular septum centred along or as near to the midline of the imaging sector as possible. This allows for a full electronic rotation around the RV apex whilst maintaining a fixed probe position. The first view at 0° shows the lateral RV wall with the left sided landmark being the mitral valve. The second view at approximately + 40° shows the anterior RV wall and the coronary sinus; thirdly at approximately − 40° the inferior RV wall and the aortic valve and lastly at approximately − 90° the inferior coronal view (CV) with the inferior wall and the anterior segment of the right ventricular outflow tract (RVOT) (Fig. 1; supplementary videos 1–4). With correct alignment and complete RV wall visualization, it is possible to perform quantitative analysis of RV function on all walls (Fig. 2). Feasibility and values of the established RV functional echo parameters, tricuspid annular plane systolic excursion (TAPSE), tissue Doppler imaging derived tricuspid annular peak systolic velocity (RV-S′) and RV wall longitudinal strain (RV-LS) were assessed retrospectively by an experienced echocardiographer (DB) on each of the 4 wall segments. TAPSE and RV-S′ parameters were deemed feasible to measure if the respective M-mode or tissue Doppler tracing was of adequate quality for the measurement to be performed accurately. In addition to the values from the individual RV walls, a multi-wall average was calculated when at least three walls from one individual were feasible to measure.

### RV speckle tracking analysis

The four RV wall datasets were digitally exported to a vendor-neutral server (TomTec Imaging Systems, Unterschleissheim, Germany) and data analysis was performed offline by one independent observer (DB), using DICOM greyscale images. To assess peak systolic global longitudinal RV strain, an RV algorithm wall motion tracking software was used (2D CPA, Image-Arena version 4.6; TomTec Imaging Systems). The endocardial border of the RV free wall and septum were manually traced at end systole and adjusted accordingly in end diastole if required. This was performed in each of the four multi-plane views previously described. A single segment strain value for the RV free wall calculated by the software is reported in this study (RV-LS). A measurement was considered feasible if all portions of the RV wall tracked accurately throughout the cardiac cycle. In cases where tracking was not accurate, attempts were made to re-adjust the endocardial border manually.

### RV 3D analysis

The 3D datasets were digitally exported to the same TomTec server and analysed retrospectively by DB using specialised RV analysis software (TomTec 4D-RV function 2.0). After placing set landmarks, RV volumes and ejection fraction (RVEF) were automatically calculated over the entire cardiac cycle. If inadequate tracking occurred the endocardial contours were manually adjusted however if this was not successful, the dataset was deemed unfeasible to measure and excluded from analysis.

### Statistical analysis

The distribution of data was assessed using histograms and the Shapiro–Wilk test. Depending on the data distribution, continuous data is presented as mean ± standard deviation (SD) or median [inter-quartile range], whilst categorical data is presented as frequencies and percentages. For comparison of normally distributed continuous variables the independent samples T-test was used and in case of skewed distribution, the Mann–Whitney U-test was applied. For comparison of frequencies the X^2^ test or Fisher’s exact test was used. For comparison of within-subject continuous parameters, the ANOVA test was used. Intra-observer agreement was assessed by repeated analysis in a random sample of ten study subjects performed at least six months after the initial analysis and blinded to the initial results. Assessment of inter-observer agreement was performed by a second observer in the same sample (JSM). The agreement between two measurements was determined as the mean of the differences + 1.96 SD. Additionally, the coefficient of variation was provided (SD of the differences of two measurements divided by their mean). All statistical analyses were performed using the Statistical Package for Social Sciences version 25 (SPSS, Inc., Armonk, NY, USA). The statistical tests were two-sided and a p-value < 0.05 was considered statistically significant.

## Results

Sixty-two surgically corrected Tetralogy of Fallot patients (median age—28 [22, 39] years, 65% male; age at surgical correction—1.1 [0.5, 3.6] years) were included in this study (Table 1). 10 further patients were screened but excluded due to poor image quality (MPE or 3D-TTE were not adequate for analyses). Detailed RV assessment by 2D multi-plane echocardiography was performed in all patients. Twenty-seven (44%) of these patients had undergone at least one pulmonary valve re-intervention (PVI) prior to echocardiography (age at PVI: 25.9 ± 12.4 years; 21 (77.7%) surgical replacement with homograft valve; 5 (18.5%) percutaneous Melody valve implantation following previous surgical intervention; 1 (3.7%) surgical valvotomy). Twenty-two (36%) patients had echocardiographic evidence of RV volume overload (≥ moderate pulmonary regurgitation), eleven (18%) with pressure overload (tricuspid regurgitation velocity ≥ 2.8 m/s), thirteen (21%) with both pressure and volume overload criteria and sixteen (25.8%) had no evidence of RV overload. Sixty-two age and gender matched healthy subjects (median age—29.7 [26.6, 35.8] years, 53% male) who were recruited for a separate study at our centre underwent the same echocardiographic imaging and were used as a control group (Table 1).

**Table 1:** Clinical and electrocardiographic characteristics of patients with repaired Tetralogy of Fallot and healthy age and gender matched controls

	Tetralogy of Fallot (n – 62)	Controls (n – 62)	p-value
Clinical			
Age (years)	28.0 [22.0, 39.0]	29.7 [26.6, 35.8]	0.43
Male, n (%)	40 (64.5)	33 (53.2)	0.20
Height (cm)	175.8 ± 11.2	175.9 ± 9.0	0.87
Weight (kg)	71.2 ± 13.5	71.8 ± 11.2	0.93
Body Mass Index (kg/m^2^)	22.9 ± 3.0	23.1 ± 2.8	0.87
Systolic blood pressure (mmHg)	122 ± 15	123 ± 12	0.67
Diastolic blood pressure (mmHg)	76 ± 11	77 ± 7	0.76
Sinus rhythm, n (%)	57 (91.9)	62 (100)	**0.021**
Heart rate (beats/min)	63 [57, 70]	60 [53, 65]	**0.043**
QRS duration (msec)	141 ± 30	96 ± 9	** < 0.001**
Age at initial surgical correction (years)	1.1 [0.5, 3.6]	–	
Trans-annular patch used, n (%)	37 (59.7)	–	
Age at pulmonary valve intervention (n = 27, years)	25.9 ± 12.7	–	
Echocardiography			
RV basal dimension (mm)	46.5 ± 9.3	39.0 ± 5.7	** < 0.001**
RV length dimension (mm)	90.5 ± 8.8	82.0 ± 7.8	** < 0.001**
RV end diastolic area (cm^2^)	34.9 ± 9.6	26.0 ± 4.8	** < 0.001**
RV end systolic area (cm^2^)	23.7 ± 8.1	15.2 ± 3.4	** < 0.001**
Fractional area change (%)	32.9 ± 8.9	41.9 ± 6.7	** < 0.001**
RV 3D end diastolic volume (ml)	195.8 [150.5, 255.1]^a^	103.0 [88.0, 120.0]^b^	** < 0.001**
RV 3D end systolic volume (ml)	102.6 [82.9, 158.7]^a^	43.0 [33.5, 51.0]^b^	** < 0.001**
Tricuspid regurgitation velocity (m/s)	2.6 [2.5, 3.0]	–	
≥ Moderate tricuspid regurgitation	4 (6.6)	–	
≥ Moderate pulmonary regurgitation	35 (57.4)	–	
≥ Moderate pulmonary stenosis	3 (4.9)	–	

Statistically significant p-values in bold (p < 0.05)

Data expressed as mean ± standard deviation; median [inter-quartile range] or number (%)

*RV* right ventricle

^a^n = 35

^b^n = 45

### Multi-plane parameters for RV function

**Fig. 3:**
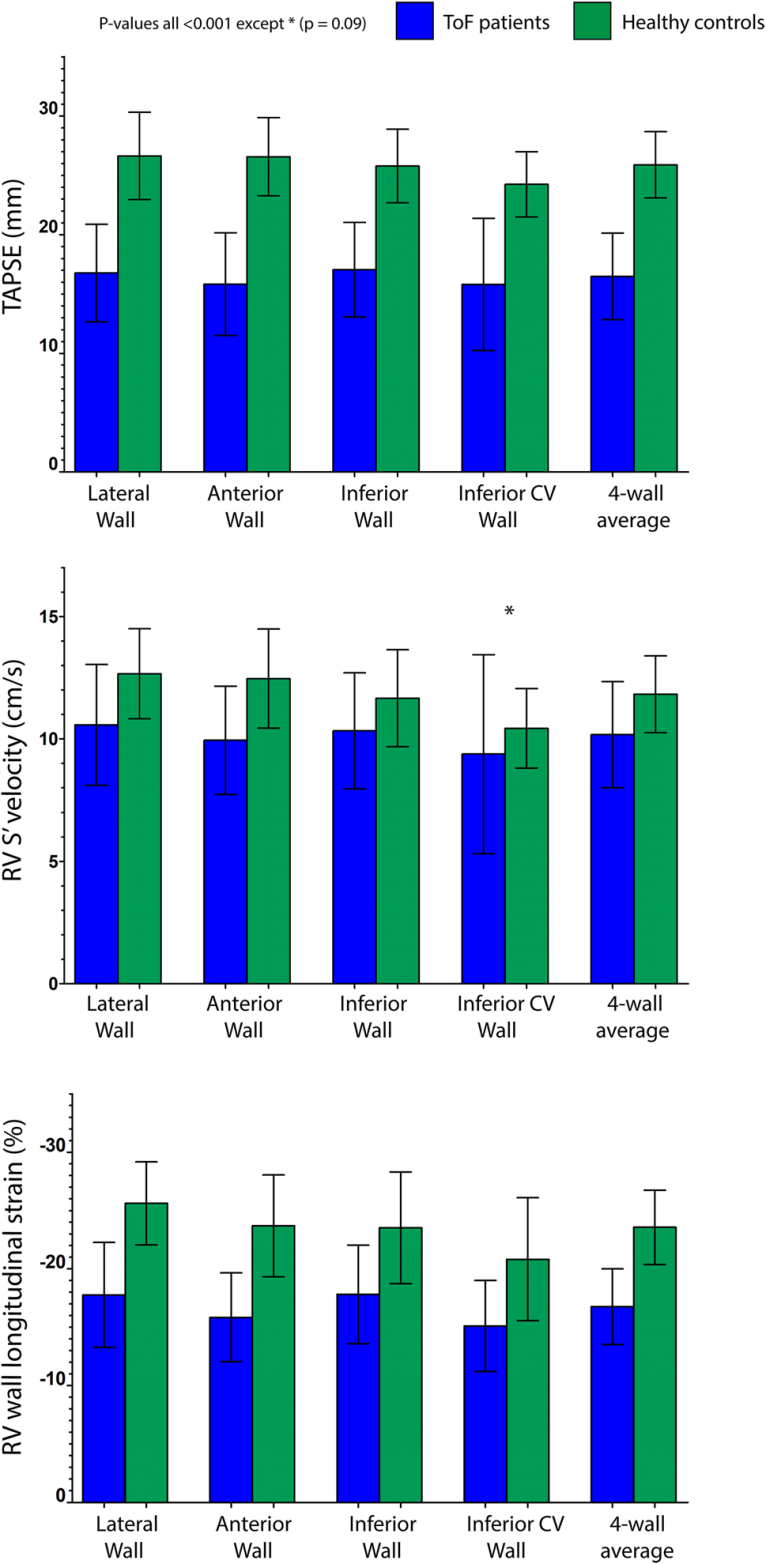
Bar charts presenting multi-plane right ventricular echocardiographic parameters in Tetralogy of Fallot (ToF) patients and in healthy controls. **A** Tricuspid annular plane systolic excursion (TAPSE); **B** tricuspid annular peak systolic velocity (RV-S′); **C** RV wall longitudinal strain (RV-LS)

Feasibility of multi-plane TAPSE and RV S′ measurements were excellent across all RV walls individually and as a multi-wall average (93.5–100%). Feasibility of RV-LS was highest in the lateral and inferior walls (95.2% and 83.9%) and there was moderate feasibility for the anterior and inferior CV walls (66.1%). An averaged value calculated from ≥ 3 RV walls was possible in 79% of cases. In comparison with the ToF group, the lateral wall feasibility was lower in controls (82.3%) but in contrast the inferior CV wall was higher (74.2%). Feasibility of 3D echo-derived RVEF in ToF patients was lower than any multi-plane measurement (56.5%), but was higher in controls (72.6%). When comparing ToF patients to normal healthy individuals, mean multi-wall averaged TAPSE was 16.5 ± 3.7 mm vs 25.9 ± 2.8 mm (p ≤ 0.001); averaged RV-S′ was 10.2 ± 2.2 cm/s vs 11.8 ± 1.6 cm/s (p ≤ 0.001) and averaged RV-LS was − 16.8 ± 3.2% vs − 23.6 ± 3.2% (p ≤ 0.001). Mean values for each segment are described in Table 2 and presented in Fig. 3. In ToF patients, the values of all three respective functional parameters were highest in the lateral and inferior walls. These differences were most pronounced in RV-LS measurements (lateral wall − 17.8 ± 4.5%; anterior: − 15.9 ± 3.8%; inferior: − 17.8 ± 4.2%; inferior CV: − 15.1 ± 3.9%, p ≤ 0.05 for lateral and inferior segments vs inferior CV). Mean 3D RVEF was 45.7 ± 7.0% in ToF patients and 59.1 ± 4.0% in controls (p ≤ 0.001). All RV dimensions and multi-plane functional parameters in ToF patients were significantly reduced compared with those of healthy controls (p ≤ 0.001 or < 0.01) with the exception of inferior CV wall RV-S′ measurement (p = 0.09).

**Table 2:** Comparison of 3D and 2D multi-plane echocardiographic functional parameters in patients with repaired Tetralogy of Fallot and healthy age and gender matched controls

	Fallot (n = 62)	Feasibility (%)	Controls (n – 62)	Feasibility (%)	p-value
RV 3D ejection fraction	45.7 ± 7.0	56.5	59.1 ± 4.0	72.6	** < 0.001**
TAPSE (mm)					
Lateral wall	16.8 ± 4.1	100.0	26.6 ± 3.7	98.4	** < 0.001**
Anterior wall	15.8 ± 4.3	96.8	26.6 ± 3.3	95.2	** < 0.001**
Inferior wall	17.0 ± 3.9	98.4	25.8 ± 3.1	98.4	** < 0.001**
Inferior CV wall	16.4 ± 3.5	93.5	24.2 ± 2.7	91.9	** < 0.001**
RV wall average	16.5 ± 3.7	100.0	25.9 ± 2.8	95.2	** < 0.001**
RV-S′ (cm/s)					
Lateral wall	10.6 ± 2.4	100.0	12.7 ± 1.8	96.8	** < 0.001**
Anterior wall	9.9 ± 2.2	98.4	12.5 ± 2.0	93.5	** < 0.001**
Inferior wall	10.3 ± 2.4	96.8	11.7 ± 2.0	93.5	**0.001**
Inferior CV wall	9.8 ± 2.2	96.8	10.4 ± 1.6	90.3	0.09
RV wall average	10.2 ± 2.2	100.0	11.8 ± 1.6	95.2	** < 0.001**
RV-LS (%)					
Lateral wall	− 17.8 ± 4.5	95.2	− 25.6 ± 3.6	82.3	** < 0.001**
Anterior wall	− 15.9 ± 3.8	66.1	− 23.7 ± 4.4	62.9	** < 0.001**
Inferior wall	− 17.8 ± 4.2	83.9	− 23.5 ± 4.8	77.4	** < 0.001**
Inferior CV wall	− 15.1 ± 3.9	66.1	− 20.8 ± 5.3	74.2	** < 0.001**
RV wall average	− 16.8 ± 3.2	79.0	− 23.6 ± 3.2	72.6	** < 0.001**

Statistically significant p-values in bold (p < 0.05)

Data expressed as mean ± standard deviation. RV wall average feasibility if ≥ 3 walls measureable (not including septum)

*RV* right ventricle, *TAPSE* tricuspid annular plane systolic excursion, *RV-S′* tricuspid annular systolic velocity by tissue Doppler imaging, *RV-LS* right ventricular wall longitudinal strain

### Comparison with 3D echocardiography

**Fig. 4:**
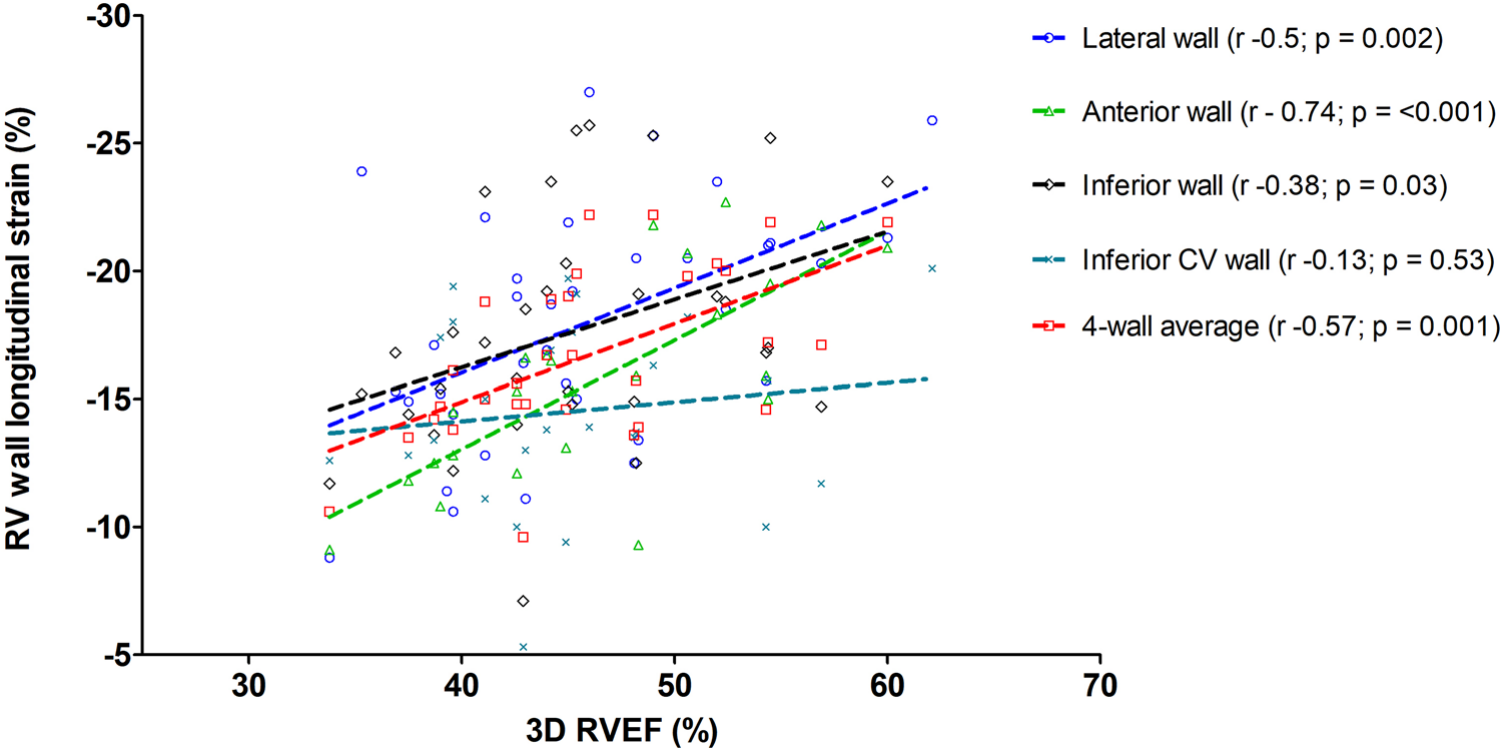
Scatter plot presenting correlations between multi-plane right ventricular wall longitudinal strain (RV-LS) and three dimensional echo-derived right ventricular ejection fraction (3D RVEF)

There were positive correlations between the multi-plane TAPSE and RV-S′ parameters and 3D RVEF (Table 3; *r* values 0.38–0.51, p ≤ 0.03). Figure 4 demonstrates positive correlations between RV-LS and 3D RVEF measurements for the lateral (*r* =  − 0.50, p = 0.002), anterior (*r* =  − 0.74, p ≤ 0.001) and inferior (*r* =  − 0.38, p = 0.03) walls and also the multi-wall averaged value (*r* =  − 0.57, p = 0.001). There was however no significant correlation with the inferior CV wall (*r* =  − 0.13, p = 0.53).

**Table 3:** Correlations between 3D right ventricular ejection fraction and 2D multi-plane echocardiographic functional parameters in patients with repaired Tetralogy of Fallot

	Pearson’s r	P-value
TAPSE (mm)		
Lateral wall	0.53	**0.001**
Anterior wall	0.41	**0.016**
Inferior wall	0.44	**0.009**
Inferior CV wall	0.51	**0.002**
Average	0.51	**0.002**
RV-S′ (cm/s)		
Lateral wall	0.48	**0.003**
Anterior wall	0.38	**0.025**
Inferior wall	0.42	**0.013**
Inferior CV wall	0.45	**0.008**
Average	0.46	**0.005**
RV-LS (%)^a^		
Lateral wall	− 0.50	**0.002**
Anterior wall	− 0.74	**0.000**
Inferior wall	− 0.38	**0.033**
Inferior CV wall	− 0.13	0.53
Average	− 0.57	**0.001**

Statistically significant p-values in bold (p < 0.05)

Average feasibility if ≥ 3 segments measureable

*TAPSE* tricuspid annular plane systolic excursion, *RV-S′* tricuspid annular systolic velocity by tissue Doppler imaging, *RV-LS* right ventricular wall longitudinal strain

^a^More negative RV-LS values indicate better function

### Impact of significant pulmonary regurgitation on multi-plane RV longitudinal strain

Thirty-five (56%) patients had echocardiographic evidence of moderate or severe PR of which six (17%) had undergone surgical PVI. The remaining twenty-seven patients had less than moderate PR although twenty-one (78%) underwent surgical PVI. In the sub-group with significant PR, RV-LS values were higher in all RV walls although none were significantly different statistically (lateral wall: − 18.4 ± 4.2 vs − 16.8 ± 4.9%; anterior: − 16.6 ± 3.7% vs − 14.8 ± 4.0%; inferior: − 18.3 ± 3.8% vs − 17.4 ± 4.7%; inferior CV: − 15.8 ± 3.7% vs − 14.4 ± 4.2%; average: − 17.4 ± 2.9% vs − 16.0 ± 3.5%; all p =  > 0.05—supplementary Fig. 6).

### Intra-observer and inter-observer agreement

**Fig. 5:**
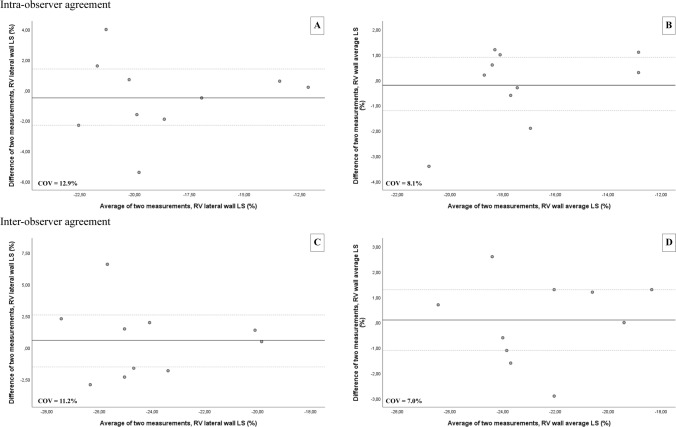
Bland–Altman plots demonstrating intra- and inter-observer agreement of RV wall longitudinal strain (RV-LS) measurement. Top panel shows intra-observer agreement for the lateral wall (**A**) and multi-wall average (**B**). Bottom panel shows inter-observer agreement for the lateral wall (**C**) and multi-wall average (**D**). The solid lines depict the mean difference of two measurements and the dashed lines depict the limits of agreement. *COV* coefficient of variation

Intra- and inter-observer agreement for RV wall longitudinal strain measurement were evaluated in a random subset of ten subjects in all four views (Table 4 and Fig. 5). The mean difference of the intra-observer measurement for each view was as follows: lateral wall, − 0.5 ± 2.5%; anterior wall, 0.4 ± 1.6%; inferior wall, 0.6 ± 1.1%; inferior CV wall, − 1.2 ± 3.0%; multi-wall average, − 0.2 ± 1.5%. The mean difference of the intra-observer measurement was: lateral wall, 0.6 ± 2.9%; anterior wall, 0.6 ± 5.0%; inferior wall, 0.1 ± 4.5%; inferior CV wall, − 1.0 ± 3.8%; multi-wall average, 0.1 ± 1.7%.

**Table 4:** Intra and inter-observer agreement for RV wall longitudinal strain measurement

	Intra-observer		Inter-observer	
	Mean difference	Coefficient of variation (%)	Mean difference	Coefficient of variation (%)
RV-LS (%)				
Lateral wall	− 0.5 ± 2.5	12.9	0.6 ± 2.9	11.2
Anterior wall	0.4 ± 1.6	10.1	0.6 ± 5.0	19.6
Inferior wall	0.6 ± 1.1	5.4	0.1 ± 4.5	19.5
Inferior CV wall	− 1.2 ± 3.0	17.3	− 1.0 ± 3.8	18.1
Average	− 0.2 ± 1.5	8.1	0.1 ± 1.7	7.0

Data expressed as mean ± standard deviation

*RV-LS* right ventricular wall longitudinal strain

## Discussion

2D MPE is a novel imaging approach for the assessment of regional and global right ventricular function in patients with repaired ToF. From one apical acoustic window, visualisation and quantification of four different RV walls (lateral, anterior and two inferior walls) can be performed using electronic plane rotation. Moreover, this technique is easily applicable in daily clinical practice when image quality is reasonable, has a short-learning curve and additional acquisition time. Quantification of RV function using 2D MPE was highly feasible and functional values correlated positively with 3D RVEF measurement. Multi-plane functional values in ToF patients are reduced compared to those of healthy individuals in all RV walls. Furthermore, there are evident RV regional differences present, namely higher values in the lateral and inferior walls compared to those of the anterior and inferior CV walls. As a result, multi-wall averaged values were reflective of lower global RV wall function when compared to the standard lateral wall value.

### Right ventricular echocardiographic assessment in repaired Tetralogy of Fallot

All multi-plane functional parameters were significantly reduced in ToF patients compared to matched healthy controls. It is reported that RV systolic function as assessed using conventional echocardiographic parameters such as TAPSE measurement is impaired in > 75% of cases [10]. Furthermore, the use of speckle tracking also reveals the prevalence of abnormalities in RV myocardial deformation [11, 12]. Of the three parameters assessed in this study, RV-LS is reported to have the strongest correlation with CMR-derived RVEF when compared to TAPSE or RV S′ measurements [13]. 74% of ToF patients included in this study had echocardiographic evidence of either pressure overload, volume overload or a combination of both. It is widely accepted that long term overload of the RV leads to progressive chamber dilatation and dysfunction, and provides one of the main risk factors for adverse cardiovascular events [4]. Notably, RV global longitudinal strain has been shown to be significantly lower in chronic pressure overload compared to volume overload [14, 15]. However, volume overload from chronic PR leads to pronounced apical broadening and thus eccentric bulging of the RV free wall [16, 17]. This change in RV geometry towards a rounded apical curvature can result in increased wall stress and reduced apical deformation [18]. For this reason, RV free wall strain progressively decreases from base to apex in ToF [11, 17, 18]. Despite using a single segment RV-LS measurement, this notion accounts for a lower average value of the entire RV wall in all multi-plane views when compared to normal RV geometry.

Detailed RV functional assessment using 2D MPE was more feasible than by 3D echo in ToF patients. In addition to superior spatial and temporal resolution [19], each 2D MPE view can be optimised by the sonographer to improve RV wall endocardial border visualisation, whether by altering probe position, angulation or instrumentation. This can also be used to overcome difficulties posed by severely dilated RV’s which 3D pyramidal datasets may fail to fully accommodate. Due to retro-sternal orientation, the RV anterior wall and RV apex are particularly affected by echo artefacts and dropout and these regions can become more difficult to track reliably with 3D.

### New insights from multi-plane RV function assessment

The basis of this study was the work carried out in our centre by McGhie et al. [6], demonstrating feasibility of this four-view multi-plane RV assessment model and proposing normal values in a healthy population. The authors reported higher mean TAPSE, RV-S′ and RV-LS values in the lateral and anterior walls compared to the inferior and inferior CV walls, albeit still within normal reported limits [8, 19, 20]. Regional differences were also observed in the present study, with the highest values of all functional parameters observed in the lateral and inferior walls. Functional values of the anterior wall were notably reduced. Since this RV wall is most proximal to the RVOT, the presence of a trans-annular patch from primary surgical reconstruction may explain reduced function in this region. This same multi-plane model has been applied in a cohort of children with repaired ToF [21], where conversely the lowest longitudinal strain values were reported in the two RV inferior walls. The inferior wall also correlated strongest with cardiac magnetic resonance (CMR) derived RVEF. In the present study, all MPE functional values correlated positively with 3D RVEF, with the exception of inferior CV wall RV-LS. LV-RV interdependence by virtue of the interlacing fibers of the interventricular septum (IVS) may account for these regional differences in RV wall function [5]. Given that the inferior CV wall lies most adjacent to the IVS, reduced or altered septal contraction could plausibly impact the deformation of this wall more than the lateral wall. Therefore, RV-LS measurement here may not be reflective of global RV function, hence the weak correlation with RVEF. Further insights of interdependence include the notion that a reduction in LV twist alters right ventricular mechanics in ToF patients [22, 23]. It has also previously been demonstrated that reduced RV strain, more than dilatation was a predictor of abnormal LV torsion, highlighting the interdependence of these deformational parameters [12].

### Limitations

This is a single centre study with many inherently associated limitations including a relatively small study group with smaller sub-groups. Due to the retrospective nature of the study, an insufficient number of patients underwent cardiac magnetic resonance imaging (cMRI) at the same time as echocardiography therefore the two imaging modalities were not able to be compared. It has however previously been demonstrated that 3D echo-derived RVEF correlates strongly with the gold-standard cMRI-derived measurement [24]. The general aim of the study was to determine the feasibility of multi-plane RV echocardiographic assessment and quantification in ToF patients and furthermore to report any regional differences in the RV walls. Interpretation of RV septal function should also incorporate indices of RV synchronicity in addition to LV deformation. Whilst this would be of particular interest in ToF patients, it was felt to be beyond the scope and aims of the present study and remains for future research to investigate. Finally, owing to its retro-sternal position, adequate visualisation of the entire anterior wall of the RVOT was only possible in a small group of the study population and was thus omitted. This region of the RV remains difficult to assess quantitatively with echocardiography. Much as for standard 2D-TTE, successful acquisition of 2D-MPE views of the RV is dependent upon reasonable image quality. Obesity, concomitant lung disease or multiple surgical interventions may affect spatial resolution, ultrasound penetration and provide sources of artefact.

### Future perspectives

The next step is to enrol patients in a multi-centre study with other specialist congenital heart disease centres to further assess this novel echocardiographic method in addition to comparison with CMR. Multi-plane RV assessment pre- and post-pulmonary valve intervention would also be of added value. This method could be extended to a broader range of other congenital pathologies involving volume or pressure overload of the right ventricle in addition to primary or secondary pulmonary hypertension. Furthermore, segmental assessment of deformation may provide more insights into the synchronicity of RV wall contraction. Multi-plane imaging of the RV is a novel method and all echocardiograms were performed by an experienced sonographer with expertise in congenital heart disease. Whilst we believe that multi-plane assessment of the right ventricle involves a short learning curve of around 20 echocardiograms, significant experience in echocardiography with attention to detail is essential to good image quality. This method enables the echocardiographer to assess RV function in greater detail and could be utilised in centres without the availability of RV 3D analysis software.

## Conclusion

This is the first study to implement 2D multi-plane echocardiography of the RV in adults with repaired Tetralogy of Fallot. Multi-plane functional quantification was highly feasible and values of TAPSE, RV-S′ and RV-LS were significantly reduced compared to healthy individuals. This novel method provides new insights into regional RV wall function in ToF patients, with higher functional values in the lateral and inferior walls compared to the anterior and inferior CV walls. Given the reduced availability of other imaging modalities, the integration of 2D MPE into routine TTE can enable a more comprehensive and quantitative assessment of RV function in ToF patients.

## Supplementary Information

Below is the link to the electronic supplementary material.

Supplementary file1 (AVI 2615 kb) **Video 1**. Echocardiographic video loop of the mitral valve view demonstrating the right ventricular lateral wall (0º electronic rotation).

Supplementary file2 (AVI 2551 kb) **Video 2**. Echocardiographic video loop of the coronary sinus view demonstrating the right ventricular anterior wall (approximately + 40º electronic rotation).

Supplementary file3 (AVI 2969 kb) **Video 3**. Echocardiographic video loop of the aortic valve view demonstrating the right ventricular inferior wall (approximately − 40º electronic rotation).

Supplementary file4 (AVI 2264 kb) **Video 4**. Echocardiographic video loop of the coronal view demonstrating the right ventricular inferior wall and the right ventricular outflow tract anterior wall (approximately − 90º electronic rotation).

Supplementary file5 (JPG 53 kb) Bar chart comparing multi-plane RV echocardiographic longitudinal strain (RV-LS) in Tetralogy of Fallot patients by severity of pulmonary regurgitation (PR). < MOD PR = less than moderate PR; ≥MOD PR = moderate or severe PR; PVI = Pulmonary valve intervention.

## Abbreviations

ToF: Tetralogy of Fallot
PR: Pulmonary regurgitation
RV: Right ventricle
LV: Left ventricle
2D TTE: Two-dimensional trans-thoracic echocardiography
MPE: Multi-plane echocardiography
TAPSE: Tricuspid annular plane systolic excursion
RV S′: Tricuspid annular peak systolic velocity
RV-LS: Right ventricular wall longitudinal strain
RVEF: Right ventricular ejection fraction
FAC: Fractional area change
CV: Coronal view
PVI: Pulmonary valve intervention
cMRI: Cardiac magnetic resonance imaging
