# Supplementary figures and images for: Multi-plane echocardiographic assessment of right ventricular function in adults with repaired Tetralogy of Fallot
Source: Int J Cardiovasc Imaging. 2021 May 18;37(10):2905–15. doi: 10.1007/s10554-021-02273-5 (PMC8494657; doi:10.1007/s10554-021-02273-5)

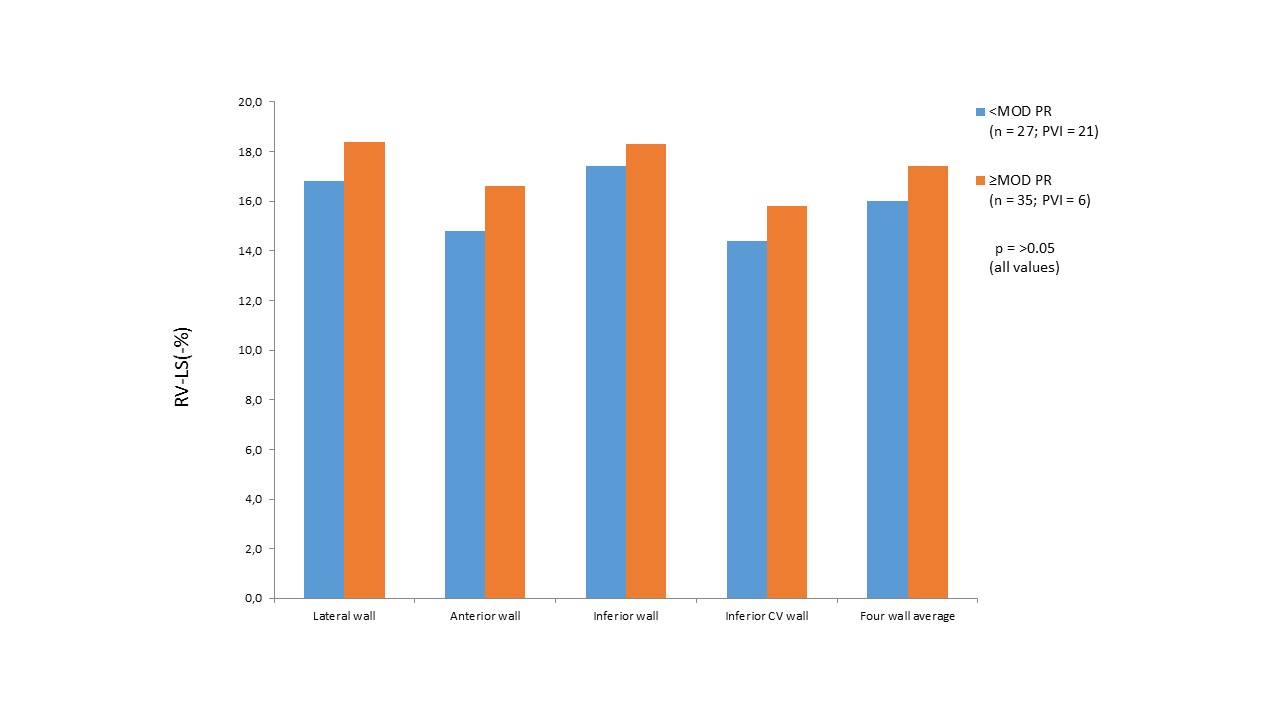

Supplement: Supplementary file 5 — Supplementary file5 (JPG 53 kb) Bar chart comparing multi-plane RV echocardiographic longitudinal strain (RV-LS) in Tetralogy of Fallot patients by severity of pulmonary regurgitation (PR). < MOD PR = less than moderate PR; ≥MOD PR = moderate or severe PR; PVI = Pulmonary valve intervention. [file 10554_2021_2273_MOESM5_ESM.jpg]
